# Growth characteristics of *Cunninghamia lanceolata* in China

**DOI:** 10.1038/s41598-022-22809-6

**Published:** 2022-10-28

**Authors:** Yangao Jiang, Zhe Hu, Zhiguang Han, Junhui Zhang, Shijie Han, Lin Hao

**Affiliations:** 1 Experimental Teaching Center, Shenyang Normal University, Shenyang, 110034 China; 2 School of Civil Engineering and Architecture, Henan University, Kaifeng, 475004 China; 3 College of Life Sciences, Qufu Normal University, Qufu, 273100 China; 4 School of Life Sciences, Henan University, Kaifeng, 475004 China; 5 College of Life Sciences, Shenyang Normal University, Shenyang, 110034 China

**Keywords:** Ecology, Ecology

## Abstract

Chinese fir (*Cunninghamia lanceolata*) is one of southern China's most important native tree species, which has experienced noticeable climate-induced changes. Published papers (1978–2020) on tree growth of Chinese fir forests in China were collected and critically reviewed. After that, a comprehensive growth data set was developed from 482 sites, which are distributed between 102.19° and 130.07°E in longitude, between 21.87° and 37.24°N in latitude and between 5 and 2260 m in altitude. The dataset consists of 2265 entries, including mean DBH (cm), mean H (m), volume (m^3^), biomass (dry weight) (kg) (stem (over bark) biomass, branches biomass, leaves biomass, bark biomass, aboveground biomass, roots biomass, total trees biomass) and related information, i.e. geographical location (Country, province, study site, longitude, latitude, altitude, slope, and aspect), climate (mean annual precipitation-MAP and mean annual temperature-MAT), stand description (origin, age, canopy density and stand density), and sample regime (plot size, number and investigation year). Our results showed that (1) the best prediction of height was obtained using nonlinear composite model Height = $1.3 + 34.23*(1 - {\text{e}}^{{\left( { - 0.01025*{\text{DBH}}^{1.347} } \right)}} )$, (R^2^ = 0.8715, p < 0.05), (2) the equation Volume = DBH^2^/(387.8 + 19,190/Height) (R^2^ = 0.9833, p < 0.05) was observed to be the most suitable model for volume estimation. Meanwhile, when the measurements of the variables are difficult to carry out, the volume model Volume = 0.03957 − 0.01215*DBH + 0.00118*DBH^2^ (R^2^ = 0.9573, p < 0.05) is determined from DBH only has a practical advantage, (3) the regression equations of component biomass against DBH explained more significant than 86% variability in almost all biomass data of woody tissues, which were ranked as total trees (97.25%) > aboveground (96.55%) > stems (with bark) (96.17%) > barks (88.95%) > roots (86.71%), and explained greater than 64% variability in branch biomass. The foliage biomass equation was the poorest among biomass components (R^2^ = 0.6122). The estimation equations derived in this study are particularly suitable for the Chinese fir forests in China. This dataset can provide a theoretical basis for predicting and assessing the potential of carbon sequestration and afforestation activities of Chinese fir forests on a national scale.

## Introduction

Forests cover c. 1/3 of the land area^1^, c. 85% of global above-ground carbon (C)^2^. Forests have considerable potential to mitigate human-caused climate change^3–6^. Afforestation and forest management are the leading measures in increasing carbon sink and mitigating greenhouse gas concentrations^7,8^. Afforestation is also an important way to restore the ecological environment in southern China^9^.

Chinese fir (*Cunninghamia lanceolata*), one of the most important native tree species in southern China, has been widely planted because of its fast growth and high-yield^10^. The Chinese fir plantation area is c. 11 million hectares, accounting for c. 12.9% of the plantation forest in China^11,12^. The carbon storage of Chinese fir forests is 63.69 Tg accounting for 1.71% of China's forests carbon storage^13^.

During the past years, Chinese fir tree growth has experienced noticeable climate-induced changes^14–17^. Therefore, understanding growth characteristics is crucial to managing and predicting Chinese fir forests under future climate change. The growth rate of Chinese fir forests is an essential indicator for evaluating the forest restoration process and carbon dioxide storage potential.

*Allom*etry, linking easily measurable variables such as diameter at breast height (DBH) and tree height (H) with other structural and functional characteristics of trees, is the most reliable and commonly used method for estimating forest biomass, volume, and net primary productivity, etc.^18–20^. There has been some synthetic research on Chinese fir in recent years. For example, the relationships: DBH, H and volume, Number of samples (N) = 182 (Location: 29°8′N, 118°24′E)^21^, N = 44 (Location: 27°45′N, 109°10′E)^22^, N = 399^23^ and N = 1840^24^ (Location: 25°16′–26°46′N 107°55′–109°36′E, Guizhou Province); DBH, H and biomass (Stems, barks, branches, leaves and roots), N = 20 (Location: 26°25′–27°04′N, 117°05′–117°40′E)^25^, N = 39 (Location: 26°25′–27°04′N, 117°05′–117°40′E)^26^; DBH, H and biomass (Stems, barks, branches, leaves, roots and total trees), N = 6 (Location: 26°28′N, 117°57′E)^27^, N = 10 (Location: 29°05′–29°23′N, 119°10′–119°20′E)^28^, N = 18 (Location: 31°10′–31°20′N, 115°30′–115°50′E)^29^; DBH, H and biomass (Stems, barks, branches, leaves), N = 600 (Location: 20°12′–34°59′N, 97°23′–122°18′E)^30^. As mentioned above, almost all the biomass investments were carried out using local observations, and the national-scale biomass equation of Chinese fir was rare. In addition, no biomass equations for roots and total trees can be used for national-scale estimates. Furthermore, the existing volume equations were all local-scale volume models, while the national-scale volume model was still unknown. To implement global forest carbon sink monitoring and assessment, developing a single tree species allometric growth equation suitable for large-scale estimation has become a trend^31^. Here, we established a comprehensive dataset of Chinese fir growth in China (N = 2265). The sample plots cover almost all the distribution areas of Chinese fir. The data of DBH and H was from the field measurement, and the data of volume and biomass was from the felled trees. In this study, national-scale allometric models were developed to estimate the DBH-Height relationships, stem volume, and biomass (Stems (with bark) plus barks, branches, foliage, aboveground, roots and total trees) for Chinese fir trees.

## Materials and methods

### Literature retrieval

Published studies (1978–2020) collected from available online full-text databases, including Academic Resource Search (https://scholar.lanfanshu.cn/), Sci-Hub Literature Search (https://sci-hub.se/). China Knowledge Resource Integrated Database (http://www.cnki.net/), Wanfang Data Knowledge Service Platform (http://www.wanfangdata.com/), Baidu Academic (https://xueshu.baidu.com/), Springer Link (http://link.springer.com/), and Docin (https://www.docin.com/). Different combinations of the keywords “Chinese fir” (or “*Cunninghamia lanceolata*”) with “DBH”, “height”, “volume”, and “biomass” were searched. We made a great effort to compile a complete growth dataset of planted and natural Chinese fir in Asia. The “Dataset” and “References cited in the dataset” are stored in Excel xlsx format. These references were published publicly, and the data in these papers were allowed to be cited. The collection and processing of data are permitted by the laws of the People's Republic of China.

### Data collection

From the literature, data was only used if all of the following criteria were met to obtain reliable growth data: Chinese fir monoculture plantations and natural forests (pure forest stand or its proportion exceeds 70%); When the study consists of multiple treatments, only data from the control treatment was selected, DBH and H were averaged from the measurement values of all trees in plots or with a random or systematic sampling method. Calculate the volume and biomass of a single tree based on the felled wood sample (allometry-derived data was excluded); The forest stands included in the dataset were limited to those that have not been disturbed by fire, pests, or recently logging; Data have undergone substantial checking, for example, a cross-check for relevant information from different sources and preliminary correlation analysis among growth variables.

Therefore, 2265 records were used to generate a comprehensive growth dataset of Chinese fir. The data set includes mean DBH (cm), mean H (m), volume (m^3^), biomass (kg) (dry weight) (stem (over bark) biomass, branches biomass, leaves biomass, bark biomass, aboveground biomass, roots biomass, total biomass). In addition, it also contains the following related information, geographical location (Country, province, study site, longitude, latitude, altitude, slope, and aspect), when geographic coordinates were not available in the original papers, Google Earth (Version: 7.3.4.8428) was applied to find longitude, latitude and altitude. Climate (mean annual precipitation-MAP and mean annual temperature-MAT), stand description (origin, age, canopy density and stand density), and sample regime (plot size, number and investigation year). These variables and their definitions, units, number, and range are listed in Table 1.

**Table 1:** Variable information in the data set.

Column code	Definition	Unit	Number	Range
ID	Unique identification number of each record	N/A	2265	1–2265
Province	Province location of study site	N/A	15	N/A
Study site	Locality name of study site	N/A	482	N/A
Latitude	Latitude of study site	°	2265	21.87°–37.24°N
Longitude	Longitude of study site	°	2265	102.19°–130.07° E
Altitude	Altitude of study site	m	2257	5–2260
Aspect	Slope direction of study site, including none (flat slope), sunny, half-sunny, shady, half-shady	N/A	804	N/A
Slope	Slope degree	°	1120	0–62
Origin	Natural or planted forests	N/A	2265	N/A
MAT	Mean annual temperature	°C	2265	11.9–28
MAP	Mean annual precipitation	mm	2265	837–2900
Age	Stand age	years	1507	1–180
Height	Mean tree height	m	2252	0.58–36.4
DBH	Mean diameter at breast height	cm	2265	0.9–69.2
Vtree	Mean tree volume (felled wood)	m^3^/tree	423 (1260 trees)	0.00039–2.0915
Vstand	Stand volume (felled wood data)	m^3^ ha^−1^	309	1.77–1185
Stems	Stems (over bark) biomass (dry weight, felled wood)	kg	406 (1287 trees)	0.01–466.05
Bark	Bark biomass (dry weight, felled wood)	kg	204 (438 trees)	0.00–57.83
Branches	Branches biomass (dry weight, felled wood)	kg	359 (1215 trees)	0.02–43.56
Leaves	Leaves biomass (dry weight, felled wood)	kg	378 (1234 trees)	0.07–19.58
Aboveground	Aboveground biomass (dry weight, felled wood)	kg	356 (1260 trees)	0.15–497.88
Root	Root biomass (dry weight, felled wood)	kg	338 (1030 trees)	0.03–79.98
Total	Total tree biomass (dry weight, felled wood)	kg	347 (1179 trees)	0.17–577.86
Density	Stand density	trees ha^−1^	1732	15–10,000
Area	Plot area	m^2^	1508	40–669,000
Plot	Plot numbers, i.e., replications	N/A	1506	1–342
Year	Investigation year	N/A	1278	1978–2020
Reference	Data sources	N/A	688	N/A

### Site and climate

**Figure 1:**
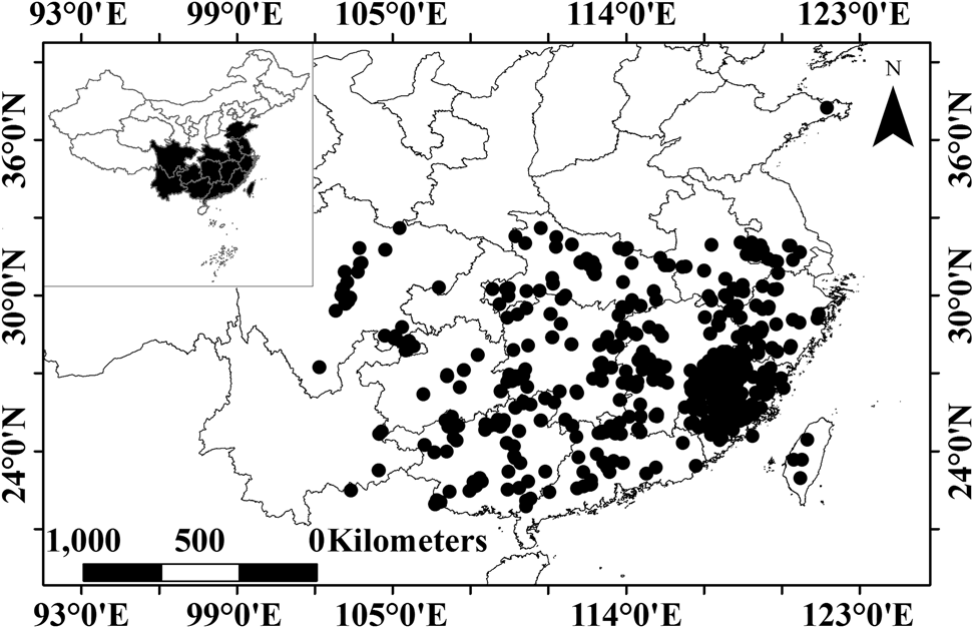
Study sites of Chinese fir in China, including Anhui, Fujian, Guangdong, Guangxi, Guizhou, Henan, Hubei, Hunan, Jiangsu, Jiangxi, Shandong, Sichuan, Taiwan, Yunnan, Zejiang provinces. The x-axis is longitude and y-axis is latitude. This figure was generated by the software QGIS 3.10.14 (https://www.qgis.org/en/site/).

The data was derived from 482 research sites in China (102.19°–130.07° E; 21.87°–37.24°N), including Anhui, Fujian, Guangdong, Guangxi, Guizhou, Henan, Hubei, Hunan, Jiangsu, Jiangxi, Shandong, Sichuan, Taiwan, Yunnan, Zhejiang provinces (Fig. 1). The average annual precipitation (MAP) ranges from 837 to 2900 mm, and the average annual temperature (MAT) ranges from 11.9 to 29 °C.

## Data estimates and evaluation

**Figure 2:**
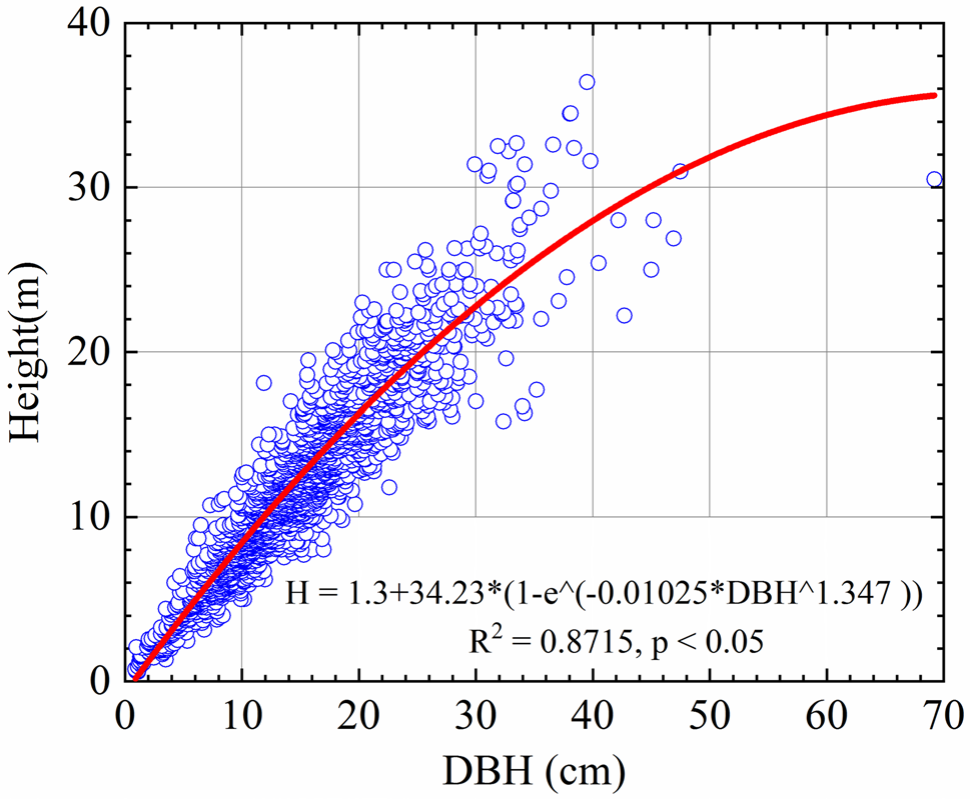
Relationship between diameter at breast height (DBH) and mean tree height (H) in the data set, Height = $1.3 + 34.23*(1 - {\text{e}}^{{( - 0.01025*{\text{DBH}}^{1.347} )}} )$ (R^2^ = 0.8715, p < 0.05).

A total of 2252 available pairs of mean DBH and mean tree height in the data set were used to establish the DBH-H correlation with a power function (${\text{H}} = 1.3 + 34.23*\left( {1 - {\text{e}}^{{( - 0.01025*{\text{DBH}}^{1.347} )}} } \right)$), R^2^ = 0.8715, P < 0.05, see Fig. 2 and Table 2). To calculate tree volume from only one known variable of DBH, tree height was firstly calculated with the power H–DBH equation in Fig. 2. The Matlab software version 2021a was used for this and subsequent analyses.

**Table 2:** Coefficient and fit statistics of different DBH-height equations.

Model no.	Equation	a	b	c	R^2^	RMSE
1	H = 1.3 + a*(DBH^b^)	0.7738	0.98		0.8585	2.074
2	H = 1.3 + (a*DBH/(b + DBH))	293.1	378.5		0.8607	2.057
3	H = 1.3 + a*(1 − e^(−b^*^DBH)^)	145.1	0.005311		0.8609	2.056
4	H = 1.3 + (DBH^2^/(a + b*DBH)^2^)	2.462	0.1341		0.8703	1.985
5	H = 1.3 + a*e^(b/DBH)^	36.02	− 16.94		0.8518	2.122
6	H = 1.3 + (a*DBH /(DBH + 1)) + b*DBH	− 0.2143	0.7403		0.8583	2.075
7	H = 1.3 + a*((DBH /(1 + DBH))^b^)	37.22	18.07		0.8551	2.098
8	H = 1.3 + a*(1 − e^(−b^*^DBHc)^)	34.23	0.01025	1.347	0.8715	1.977
9	H = 1.3 + (DBH ^2^/(a + b*DBH + c*DBH^2^))	4.967	0.7804	0.01505	0.8705	1.983
10	H = 1.3 + a*DBH*(e^(−b^*^DBH)^)	0.7714	0.002671		0.861	2.055
11	H = 1.3 + a*DBH + b*DBH^2^	0.7735	− 0.0021		0.8612	2.053
12	H = 1.3 + a*e^((b/DBH) +c)^	2.223	− 7.68	2.234	0.683	3.103
13	H = 1.3 + a*e^(−(b/DBHc))^	177	7.819	0.3849	0.8699	1.988

**Figure 3:**
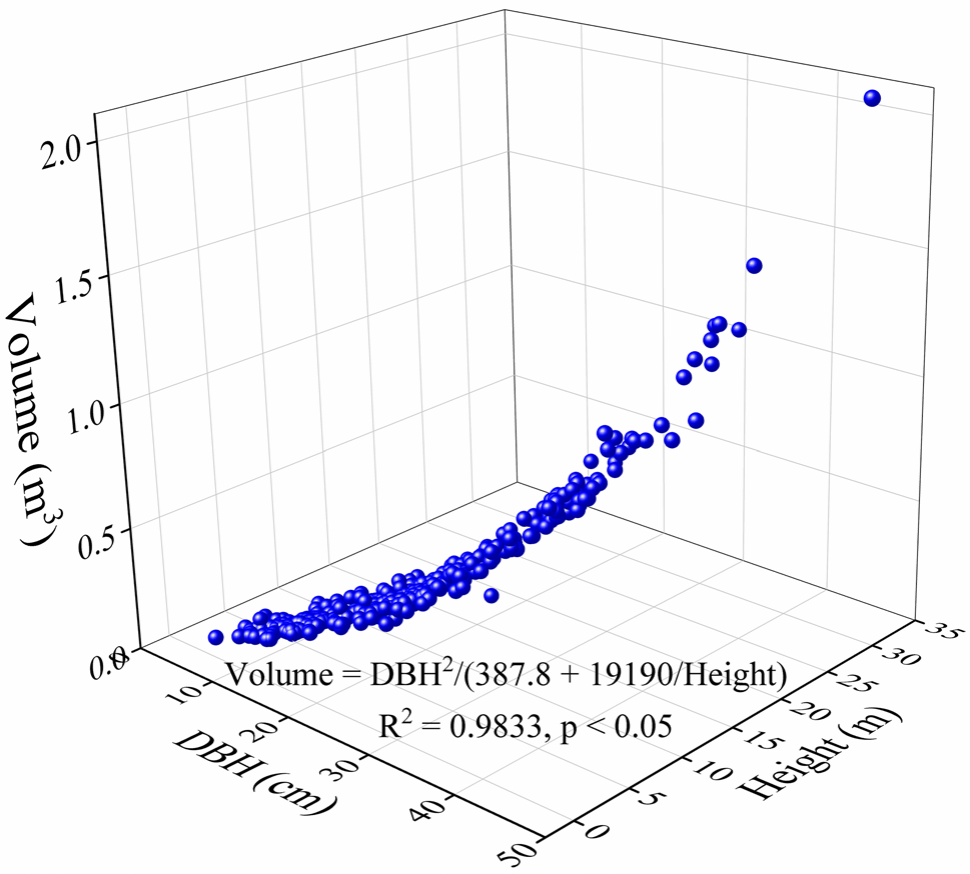
Relationships of Volume against diameter at breast height (DBH) and Height, Volume = DBH^2^/(387.8 + 19,190/Height) (R^2^ = 0.9833, p < 0.05).

The volume equations were classified as two types by the independent variables: volume = f(DBH) and volume = f(DBH, height). These equations have been previously applied for tree volume models in forestry^32–35^. There were three equations in f(DBH) and four equations in f(DBH, height), and the parameter estimates and fit statistics were computed by all equations (Table 3). Equation (7) was evaluated as the most suitable volume equation with the highest coefficient of determination (R^2^) and lowest root mean square error (RMSE) (Fig. 3). The estimated stand volume was determined by multiplying the optimal estimated tree volume by the stand density.

**Table 3:** Coefficient and fit statistics of different stem volume equations.

Model no	Equation	a	b	c	d	R^2^	RMSE	DBH range (cm)
1	V = a + bDBH^2^	− 0.06187	0.00087			0.9489	0.05274	3.6–47.5
2	V = a + bDBH + cDBH^2^	0.03957	− 0.01215	0.00118		0.9573	0.04822	3.6–47.5
3	V = aDBH + bDBH^2^	− 0.00783	0.001075			0.9562	0.04882	3.6–47.5
4	V = a + bDBH^2^H	0.02093	3.37e−05			0.9788	0.03399	3.6–47.5
5	V = aDBH^2^H	3.513e−05				0.9733	0.03808	3.6–47.5
6	V = a + bDBH^c^H^d^	79.22	− 79.4	− 0.00082	− 0.00106	0.3421	0.1892	3.6–47.5
7	V = DBH^2^/(a + b/H)	387.8	19,190			0.9833	0.03017	3.6–47.5

**Figure 4:**
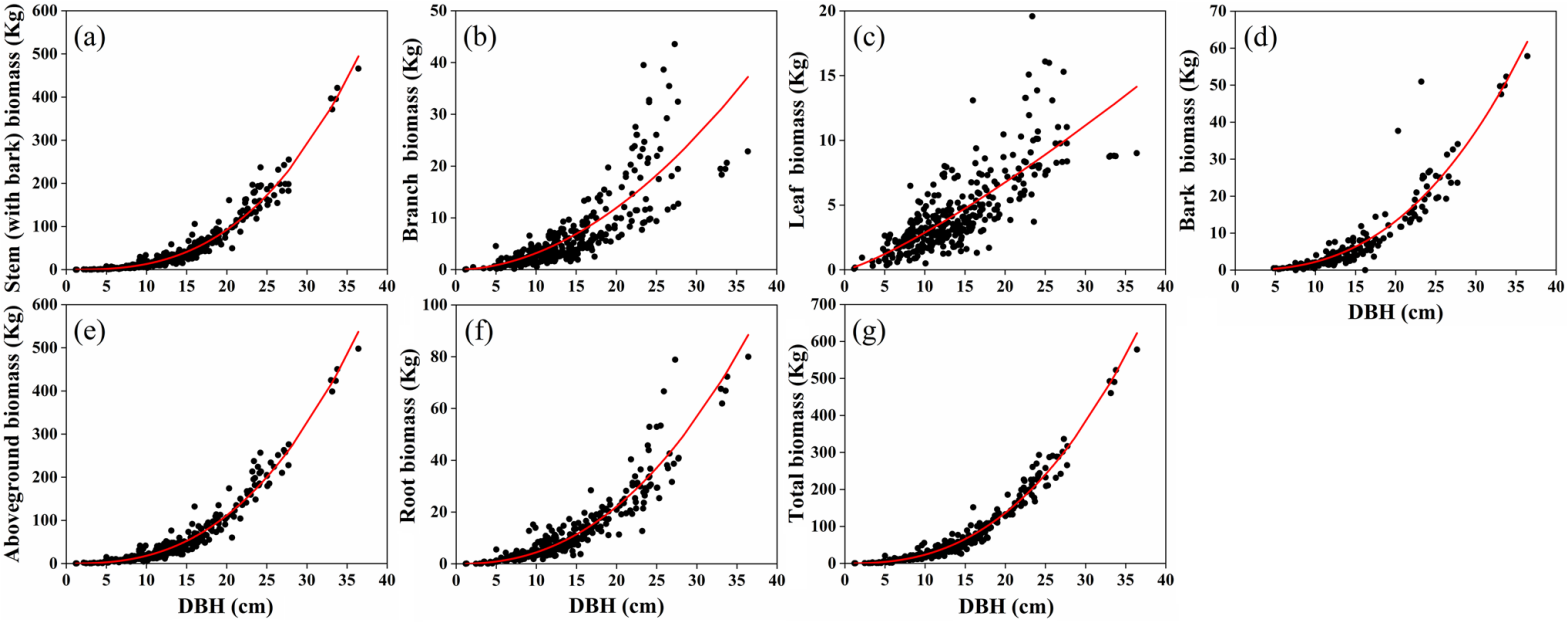
Relationships of component biomass against diameter at breast height. (**a**–**g**) Stand for stem (with bark), total branch, total foliage, aboveground, total root, and total biomass, respectively.

There is no universally accepted equation form for biomass prediction. Most biomass equations in scientific literature adopt the power function of M = aD^b^^36–39^, and this biomass model was also used in this paper. The regression equations of component biomass against DBH explained more significant than 86% variability in almost all biomass data of woody tissues, including total trees, aboveground, roots, stems (with bark), barks, and explained greater than 64% variability in branches biomass (Table 4; Fig. 4). The foliage biomass equation was the poorest among biomass components (R^2^ = 0.6122) (Table 4; Fig. 4).

**Table 4:** Allometric equations relating biomass components (kg) to diameter at breast height (DBH, cm).

Component	a	b	R^2^	RMSE	DBH range (cm)
Stems (with bark)	0.02106	2.8	0.9617	12.91	1.31–36.41
Barks	0.006084	2.566	0.8895	3.556	4.8–36.41
Branches	0.04026	1.9	0.6481	4.57	1.19–27.3
Leaves	0.1681	1.233	0.6122	1.913	1.19–27.3
Aboveground	0.04305	2.624	0.9655	13.87	1.31–36.41
Roots	0.02184	2.311	0.8671	5.259	1.31–36.41
Total trees	0.06986	2.53	0.9725	15.29	1.19–36.41

## Discussion

DBH–height relationship is widely used to estimate timber volume, biomass, and other important parameters for forest growth and yield in forest management^40^. Accurate tree height prediction is critical in forest modeling, inventory and management decision making^41^. Our results show that DBH and tree height are well correlated at the national scale, and the DBH-height model displayed a good fit (R^2^ = 0.8715, p < 0.05). Therefore, such national-scale relationships will avoid much cost of constructing site-specific relations^42^. Furthermore, it is especially useful to improve stand volume and carbon stock estimation in national-scale forest inventory.

The R^2^ value (R^2^ = 0.9833, p < 0.05) for our national-scale two-variable volume equation was similar to other local scale volume equations reported by Li^21^ (R^2^ = 0.9820), Zeng et al.^23^ (R^2^ = 0.9994), and Xia et al.^24^ (R^2^ = 0.9868). Models that incorporate DBH and height usually give good-fits^43–46^. Although the best-fit model to estimate stem wood volume was a two-variable volume equation (Table 3), in some cases, this two-variable model is not practical because the measurements of these variables are difficult to carry out with high accuracy, particularly in closed forests^47^. Besides, the results of this study verified that a one-variable equation with DBH only can also be used to get good estimates of Chinese fir tree volume in China (R^2^ = 0.9573, p < 0.05). Meanwhile, the DBH is easy to measure accurately in the field, so this one-variable equation has a practical advantage.

We developed a set of biomass equations for Chinese fir trees growing in China. In the previous studies, Li and Zhao^30^ used 600 Chinese fir trees for aboveground biomass equations, while the biomass dataset used in our study consisted of 1525 Chinese fir trees. A larger sample size can reduce parameter estimation uncertainty^48^. Compared with the previous equations^30^ developed for Chinese fir forests on a national scale, the new system of equations included two more biomass components-roots and total trees (Table 4). Belowground biomass comprises about 25% of total biomass in forest ecosystems^49^. However, because of the laborious and time-consuming^50^, direct measurements of belowground biomass are seldom^51^. Precise quantification of underground carbon storage in forest ecosystems is of great significance for effectively predicting how future environmental changes will affect global carbon dynamics^52^. There is an urgent need to develop some algorithm for estimating this carbon pool. Correlations found in this study indicate that equations using dbh as the predictive variable can offer a good estimate of the stems, total aboveground, roots, and total trees biomass, but a poor estimate of branches and foliage biomass (Table 4), which is consistent with other findings that a single DBH-based allometric equation provides a reasonable prediction for total aboveground^53,54^, root^55^, and total tree biomass^56^. The improved predictability of total and aboveground biomass may also be attributable to the fact that most biomass exists within the stem component that is, in itself, highly correlated with dbh. Poor predictions for branches and foliage biomass may result from variation in biomass allocation due to soil conditions^57^, stand age^58^ and stand density^59^, which is consistent with the previous studies^57,60,61^.

## Conclusions

In this study, 13 DBH-Height models were tested on trees in Chinese-fir forests between 1 and 180 years old in southern China. Model selection was based on goodness of fit. The best prediction of height was obtained using nonlinear composite model H = $1.3 + 34.23*(1 - {\text{e}}^{{( - 0.01025*{\text{DBH}}^{1.347} )}} )$, R^2^ = 0.8715, P < 0.05, which used three parameters, this was recommended for Chinese-fir forests in China.

We also used seven models to test suitability for Chinese fir tree volume estimation. Of these, the equation Volume = DBH^2^/(387.8 + 19,190/Height) (R^2^ = 0.9833, p < 0.05) was observed to be the most suitable model for volume estimation. In addition, when the measurements of the variables are difficult to carry out, the volume model (Volume = 0.03957 − 0.01215*DBH + 0.00118*DBH^2^) is determined from DBH only has a practical advantage.

In addition, the regression equations of component biomass against DBH explained more significant than 86% variability in almost all biomass data of woody tissues, which were ranked as total trees (97.25%) > aboveground (96.55%) > stems (with bark) (96.17%) > barks (88.95%) > roots (86.71%), and explained greater than 64% variability in branch biomass. The foliage biomass equation was the poorest among biomass components (R^2^ = 0.6122).

As mentioned above, these estimation equations derived in this study are particularly suitable for the Chinese fir forests in China.

## Supplementary Information

Supplementary Information.

## Data Availability

The datasets used and/or analysed during the current study are available from the corresponding author upon reasonable request.
